# Editorial: Oxidative stress and inflammation in type 2 diabetes: pathways to organ complications

**DOI:** 10.3389/fcdhc.2026.1976753

**Published:** 2026-09-09

**Authors:** Toshiki Otoda, Teruki Miyake, Shusuke Yagi, Ken-ichi Aihara, Keizo Kanasaki

**Affiliations:** 1 Division of General Medicine, Department of Internal Medicine, Nihon University School of Medicine, Tokyo, Japan; 2 Department of Lifestyle-related Medicine and Endocrinology, Ehime University Graduate School of Medicine, Toon, Japan; 3 Department of Community and Family Medicine, Tokushima University Graduate School of Biomedical Sciences, Tokushima, Japan; 4 Department of Internal Medicine, Anan Medical Center, Tokushima, Japan; 5 Faculty of Medicine, Internal Medicine I, Shimane University, Izumo, Japan

**Keywords:** diabetic complications, inflammation, mitochondrial dysfunction, oxidative stress, type 2 diabetes mellitus

Type 2 diabetes mellitus (T2DM) is increasingly recognized not simply as a disorder of glucose metabolism, but as a systemic disease in which metabolic stress, oxidative stress, inflammation, hypoxia, and mitochondrial dysfunction interact to promote progressive tissue injury. This Research Topic, *Oxidative Stress and Inflammation in Type 2 Diabetes: Pathways to Organ Complications*, brings together ten articles spanning molecular mechanisms, circulating biomarkers, nutritional and environmental factors, pharmacological interventions, and clinically evident complications. Collectively, these contributions illustrate how distinct pathways converge to connect metabolic dysfunction with organ injury and highlight opportunities for mechanism-based prevention and treatment.

Inflammatory regulation is an important component of this network. Alrasheed et al. synthesized evidence from 40 randomized controlled trials involving 6,029 participants and showed that glucagon-like peptide-1 receptor agonists (GLP-1RAs) reduced major inflammatory biomarkers, particularly C-reactive protein and tumor necrosis factor-α, supporting anti-inflammatory effects beyond glycemic control. Complementing this therapeutic perspective, Tleumagambetova et al. examined the interleukin-1β/interleukin-1 receptor antagonist balance in newly diagnosed adult-onset diabetes and found lower circulating interleukin-1 receptor antagonist levels and a higher pro-/anti-inflammatory ratio in islet autoantibody-positive individuals, emphasizing immunometabolic heterogeneity at disease onset.

Hypoxia-related signaling provides another link between metabolic stress and cellular adaptation. In one systematic review and meta-analysis, Su et al. examined peripheral blood hypoxia-inducible factor-1α (HIF-1α) in relation to pancreatic β-cell dysfunction and insulin resistance, finding associations between increased circulating HIF-1α and adverse metabolic indices. In a separate meta-analysis, Su et al. extended this perspective to HIF-1α, sirtuin 1, and vascular endothelial growth factor in T2DM and diabetic complications, further highlighting altered hypoxic and vascular adaptive responses in diabetes. Together, these studies underscore the need to distinguish circulating markers from tissue-specific signaling when interpreting adaptive responses to metabolic and hypoxic stress.

At the cellular level, mitochondrial dysfunction is an important intersection between metabolic stress and organ injury. Tamaki et al. investigated the role of bone morphogenetic protein 4 (BMP4) in diabetic nephropathy and demonstrated an association between BMP4 stimulation, Parkin downregulation, and impaired mitochondrial homeostasis in mesangial cells. ALK inhibition and Parkin overexpression ameliorated several mitochondrial abnormalities, extending the potential role of BMP4 beyond extracellular matrix production in mitochondrial quality control.

Other studies have highlighted potential modifiable determinants of oxidative injury. Kubo et al. compared pemafibrate to eicosapentaenoic acid in patients with T2DM and hypertriglyceridemia. Pemafibrate produced a greater reduction in malondialdehyde-modified low-density lipoprotein, whereas urinary 8-hydroxy-2′-deoxyguanosine and high-sensitivity C-reactive protein did not differ significantly between treatments. These findings illustrate the biological heterogeneity of oxidative stress and suggest that caution should be exercised when interpreting a single oxidative biomarker as representative of the entire redox state. From a nutritional perspective, Wang et al. applied machine-learning approaches to dietary oxidative balance and identified vitamin E, magnesium, and β-carotene among factors associated with urinary albumin-to-creatinine ratio-defined renal impairment, with exploratory experiments implicating AKT-related signaling.

The clinical relevance of these interconnected pathways has been reflected in studies of established complications. Rashwan et al. identified smoking, previous ulceration or amputation, and advanced chronic kidney disease as factors strongly associated with the loss of protective sensation in 387 patients with diabetic peripheral neuropathy. Zhang et al. explored the environmental dimension and reported interactions between temperature, humidity, and tongue color in diabetes, particularly among individuals with cardiovascular complications, suggesting that environmental stress may interact with impaired microcirculatory and vascular adaptations.

Finally, Srithongchai et al. reviewed the advanced glycation end-product (AGE)-receptor for the AGE (RAGE) axis as a pathogenic signaling network in diabetic complications. This review highlights membrane-bound RAGE signaling as an interface connecting carbonyl and oxidative stress with inflammatory activation, whereas soluble RAGE isoforms may provide counter-regulatory decoy activity. Importantly, the physiological functions of RAGE include the selective modulation of pathogenic signaling rather than indiscriminate receptor inhibition.

## Conclusion

Taken together, the ten contributions in this Research Topic portray diabetic complications as products of an interconnected network rather than a single pathogenic pathway. Metabolic overload, oxidative and carbonyl stress, inflammation, hypoxic responses, mitochondrial dysfunction, environmental influences, and impaired adaptive mechanisms interact across different tissues and disease stages. Simultaneously, the diversity of biomarkers and interventions examined here emphasizes that changes in individual molecular markers should not be interpreted in isolation.

Therefore, future research should seek to define the molecular switches that determine whether stress responses remain adaptive or become pathogenic, integrate complementary biomarkers across biological compartments, and establish whether selective modulation of these interconnected pathways can translate into meaningful prevention of organ injury. Such approaches, together with treatment strategies targeting oxidative stress and inflammation, may help move the management of T2DM beyond glycemic control toward mechanism-based prevention and treatment of complications.

